# Supplementary material for: Real-World Impact of SABR on Stage I Non-Small-Cell Lung Cancer Outcomes at a Scottish Cancer Centre
Source: Cancers (Basel). 2023 Feb 23;15(5):1431. doi: 10.3390/cancers15051431 (PMC10000454; doi:10.3390/cancers15051431)
Supplement: Supplementary file 1 [file cancers-15-01431-s001.zip › cancers-2124885-supplementary.pdf]

Supplemental Table S1. Stage distribution of patients with NSCLC in NHS Lothian during the three study time periods (A: 2012-2103, Pre-SABR, B: 2104-2106, Introduction of SABR, C: 2017-2019, SABR Established).

**Stage**	**Time Period**		
	**A**	**B**	**C**
**I**	24%	25%	26%
**II**	8%	9%	8%
**III**	21%	21%	22%
**IV**	46%	46%	43%

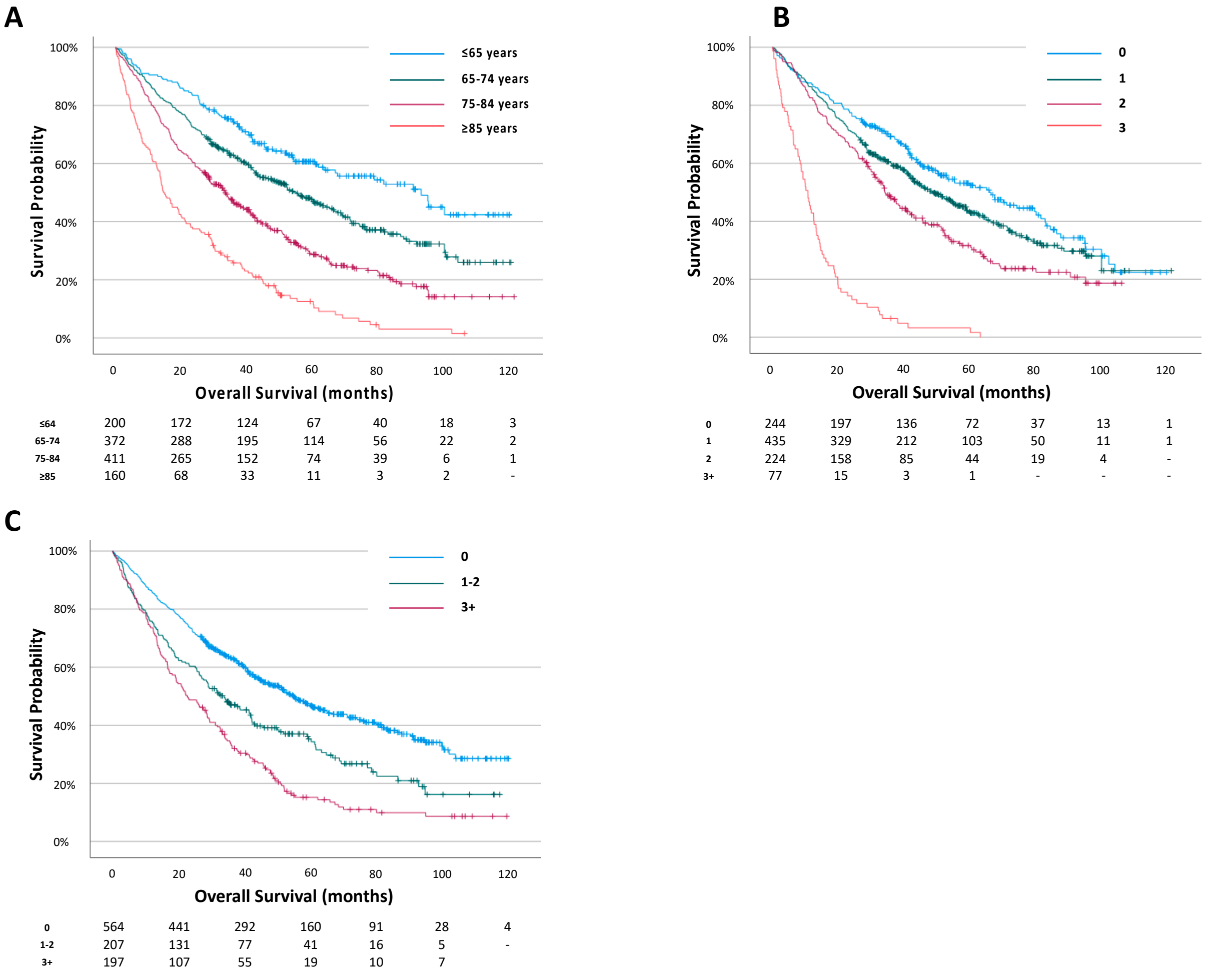

Supplemental Figure S1. Overall survival of patients with stage I NSCLC by A: Age, B: Performance Status, C: Charlson Comorbidity Index.

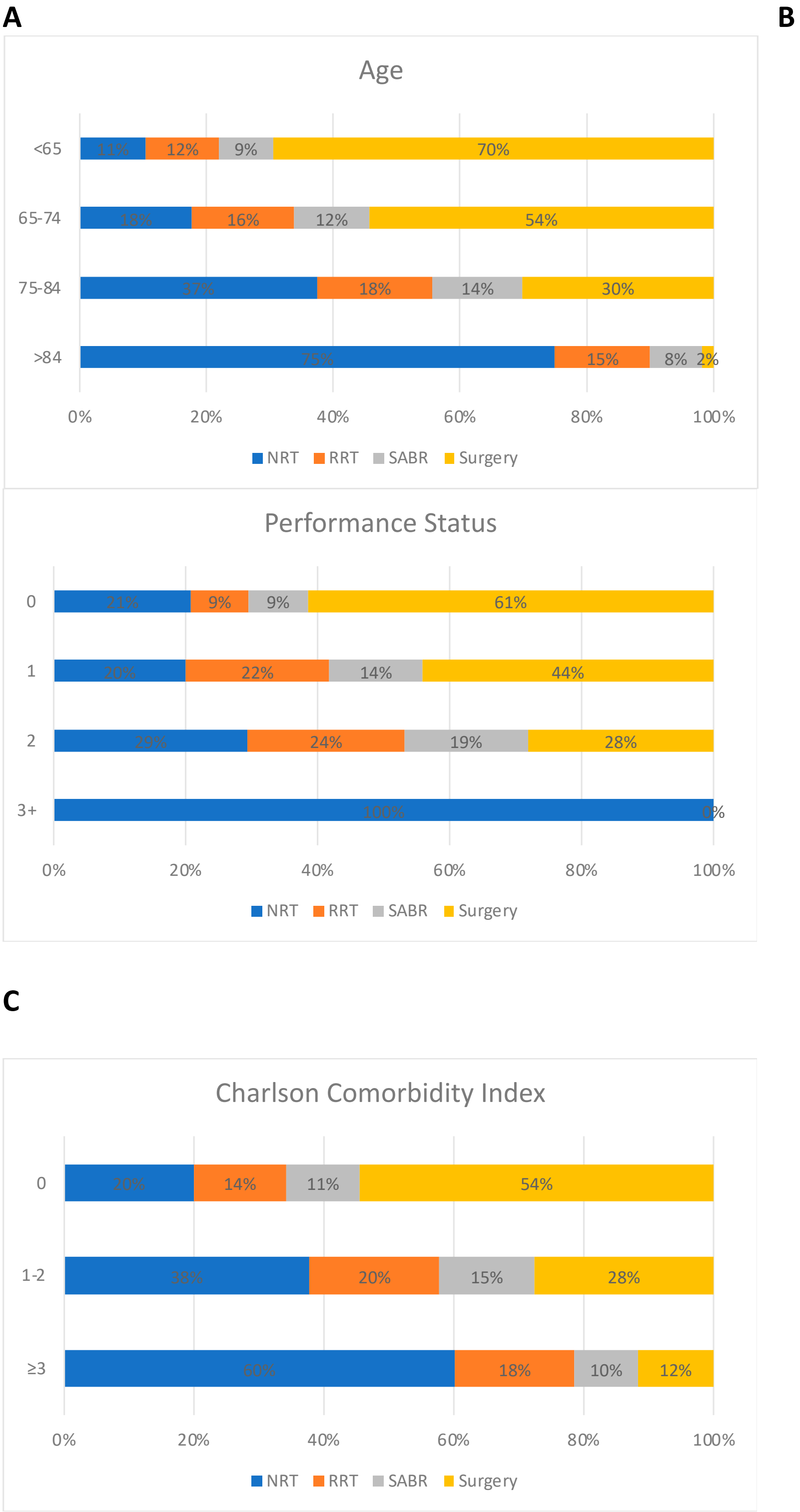

Supplemental Figure S2. Treatment utilisation for all patients with stage I NSCLC by A: Age, B: Performance Status, C: Charlson Comorbidity Index.

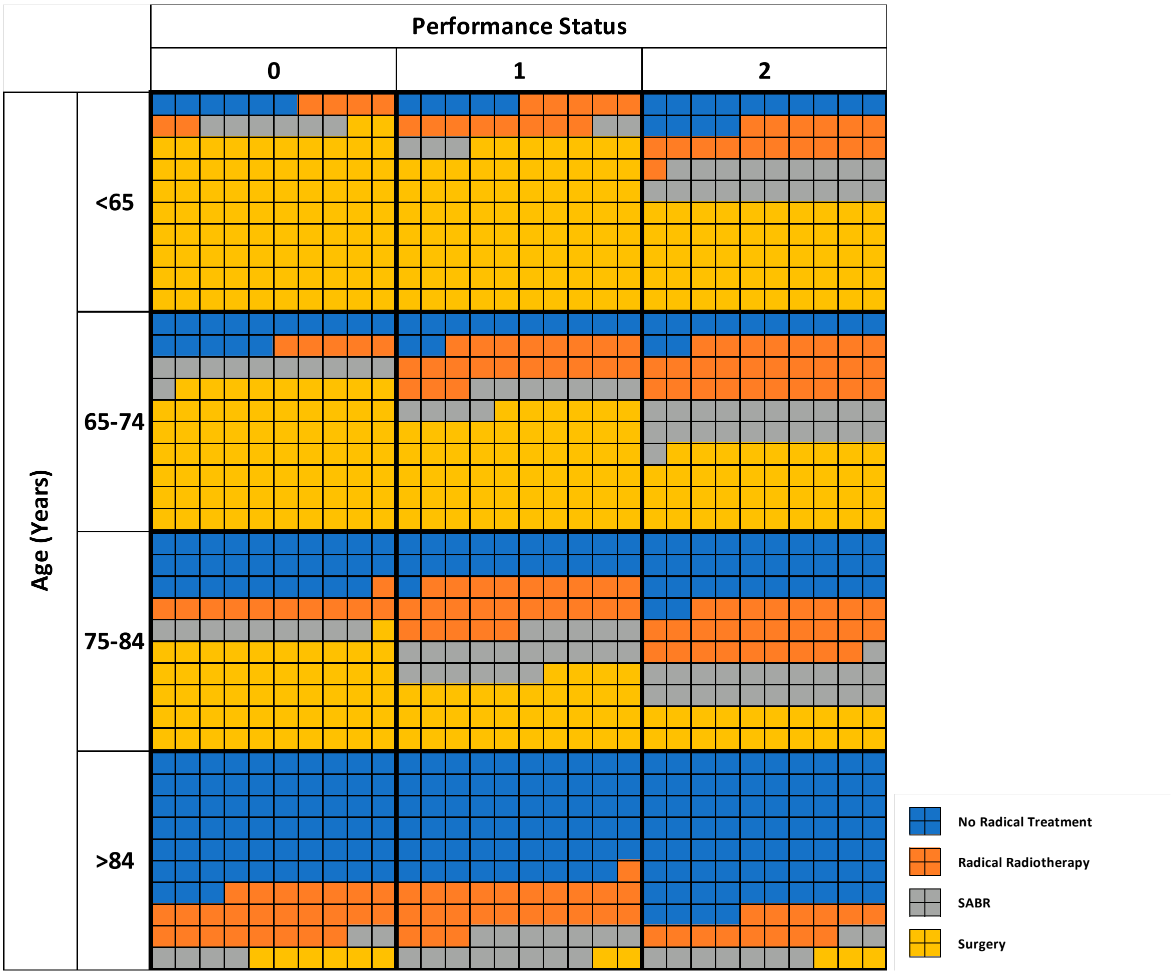

Supplemental Figure S3. Treatment choice matrix by age and performance status for all patients with stage I NSCLC.

Supplemental Table S2. Life table for overall survival of all patients with stage I NSCLC by treatment modality. (see also Figure 1).

Overall Survival (months)	0	20	40	60	80	100	120
**Surgery**	468	415	312	183	108	43	5
**SABR**	132	116	63	26	9	-	-
**CRRT**	182	132	75	32	15	4	-
**NRT**	361	130	54	25	6	1	1

Supplemental Table S3: Life tables for overall survival estimates for all patients with stage I NSCLC, and for each treatment group, by treatment time period, A: All patients, B: No radical therapy, C: Conventional Radical Radiotherapy, D: SABR, E: Any Radical Radiotherapy, F: Surgery. (see also Figure 2).

A

Overall Survival (months)	0	20	40	60	80	100	120
A – 2012/13, Pre-SABR	252	158	114	80	62	48	6
B – 2014/16, Introduction of SABR	443	305	217	168	76	-	-
C – 2017/19, SABR Established	448	330	173	18	-	-	-

B

Overall Survival (months)	0	20	40	60	80	100	120
A – 2012/13, Pre-SABR	83	24	12	6	1	1	1
B – 2014/16, Introduction of SABR	144	52	25	17	5	-	-
C – 2017/19, SABR Established	134	54	17	2	-	-	-

C

Overall Survival (months)	0	20	40	60	80	100	120
A – 2012/13, Pre-SABR	51	33	22	10	7	4	-
B – 2014/16, Introduction of SABR	69	54	35	20	8	-	-
C – 2017/19, SABR Established	62	45	18	2	-	-	-

D

Overall Survival (months)	0	20	40	60	80	100	120
A – 2012/13, Pre-SABR	-	-	-	-	-	-	-
B – 2014/16, Introduction of SABR	50	41	29	23	9	-	-
C – 2017/19, SABR Established	82	75	34	3	-	-	-

E

Overall Survival (months)	0	20	40	60	80	100	120
A – 2012/13, Pre-SABR	51	33	22	10	7	4	-

**B – 2014/16, Introduction of SABR**	119	95	47	64	43	17	-
**C – 2017/19, SABR Established**	144	120	52	5	-	-	-

F

**Overall Survival (months)**	**0**	**20**	**40**	**60**	**80**	**100**	**120**
**A – 2012/13, Pre-SABR**	118	101	80	64	54	43	5
**B – 2014/16, Introduction of SABR**	180	158	128	108	54	-	-
**C – 2017/19, SABR Established**	170	156	104	11	-	-	-

Supplemental Table S4. Overall survival estimates for all patients with stage I NSCLC, and for each treatment group, by treatment time period (A: 2012-2103, Pre-SABR, B: 2104-2106, Introduction of SABR, C: 2017-2019, SABR Established).

	**Time Period**	**Overall Survival (months) (median (IQR))**	**HR (95%CI), p**
**All Patients**	**A**	32.5 (13.0-74.8)	1 (Ref)
	**B**	38.8 (15.7-not reached)	0.87 (0.72-1.03), 0.112
	**C**	48.8 (19.0-not reached)	***0.85 (0.77-0.94), 0.002***
**No Radical Therapy**	**A**	12.7 (5.1-29.8)	1 (Ref)
	**B**	13.9 (5.0-29.3)	0.84 (0.64-1.10), 0.207
	**C**	13.4 (5.6-31.8)	0.95 (0.83-1.10), 0.513
**CRRT**	**A**	29.4 (14.4-57.4)	1 (Ref)
	**B**	41.9 (23.2-65.5))	0.81 (0.55-1.18), 0.269
	**C**	39.1 (19.2-55.6)	0.93 (0.74-1.17), 0.517
**SABR**	**A**	n/a	1 (Ref)
	**B**	46.2 (27.9-82.3)	n/a~
	**C**	not reached (31.9-not reached)	n/a
**Any Radiotherapy**	**A**	29.4 (14.4-57.1)	1 (Ref)
	**B**	42.2 (24.2-78.1)	***0.70 (0.49-0.99), 0.045***
	**C**	50.4 (24.6-not reached)	***0.75 (0.61-0.91), 0.004***
**Surgery**	**A**	68.2 (28.5-not reached)	1 (Ref)
	**B**	87.5 (35.7-not reached)	0.83 (0.60-1.13), 0.238
	**C**	Not reached (54.7-not reached)	***0.69 (0.56-0.86), <0.001***

(NRT: No Radical Treatment, CRRT: Conventional Radical Radiotherapy, SABR: Stereotactic Ablative Body Radiotherapy)

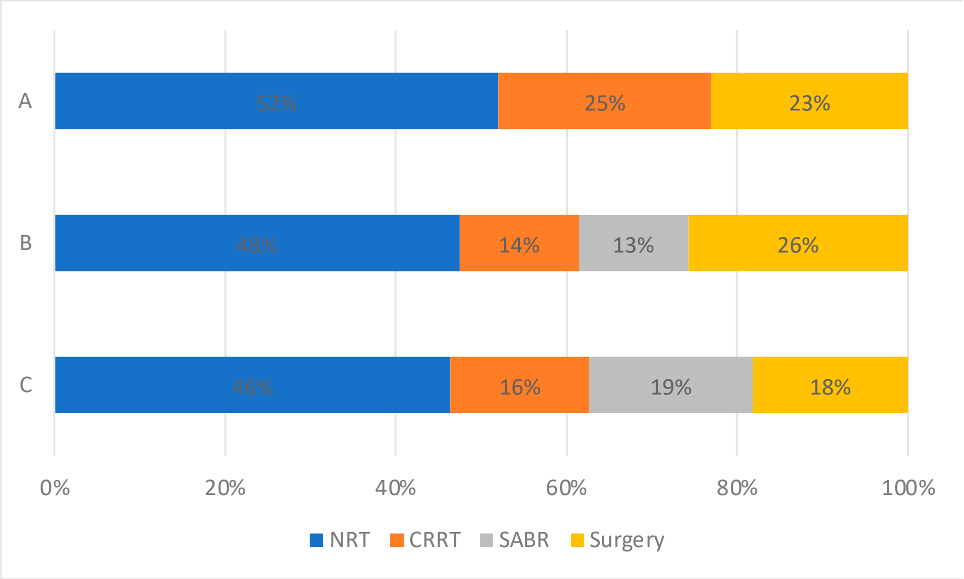

Supplemental Figure S4. Treatment utilisation for all patients aged 75 years and over with stage I NSCLC by time period.
